# Supplementary material for: Bilosomes and Biloparticles for the Delivery of Lipophilic Drugs: A Preliminary Study
Source: Antioxidants (Basel). 2023 Nov 21;12(12):2025. doi: 10.3390/antiox12122025 (PMC10741235; doi:10.3390/antiox12122025)
Supplement: Supplementary file 1 [file antioxidants-12-02025-s001.zip › antioxidants-2701543-supplementary.pdf]

antioxidants

Article

Bilosomes and Biloparticles for the Delivery of Lipophilic Drugs: A Preliminary Study

Maddalena Sguizzato [1,†], Francesca Ferrara [1,†], Nada Baraldo [1], Agnese Bondi [1], Annunziata Guarino [2], Markus Drechsler [3], Giuseppe Valacchi [4,5,6] and Rita Cortesi [1,7,*]

Table S1. Composition of the produced biloparticles expressed as percentage by weight.

Acronym	Tristearin	Miglyol 812	Bile acid	Budesonide	Poloxamer 188 2,5%w/w water solution
BilopS	4.2	-	0.8	-	95
BilopSB	4.1	-	0.8	0.1	95
BilopN	2.55	1.65	0.8	-	95
BilopNB	2.45	1.65	0.8	0.1	95

Table S2. Composition of ursodeoxycholic bilosomes used for the preformulatory study

Component	Concentration mg/ml	
	BiloPU	BiloPCU
PC	20	20
CH	-	2.5
UDCA	5	2.5

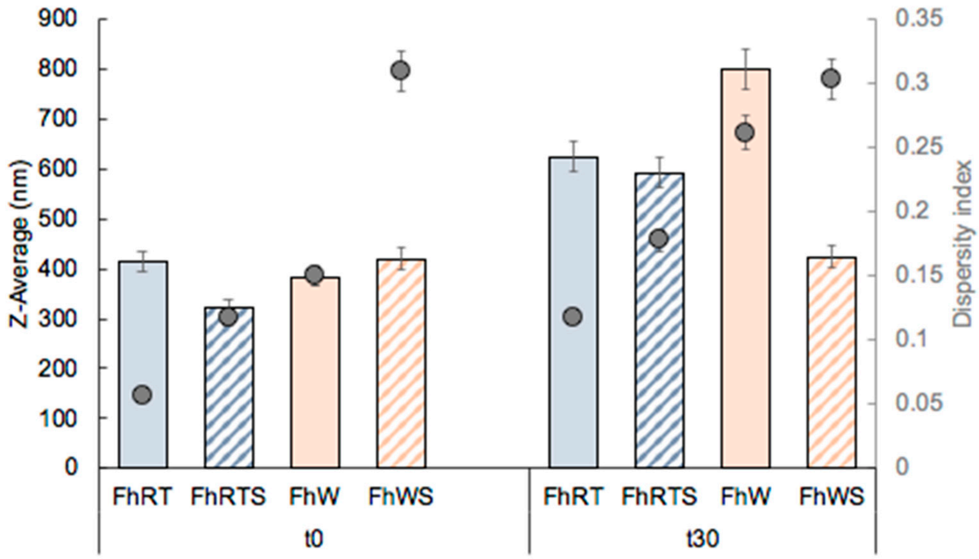

Figure S1. Size after production and after 30 days of storage of Bilos_PCU obtained following different protocols (FhRT, FhRTS, FhW, FhWS). The size is expressed as average diameter (Z-Average) and dispersity index (PdI) . The values are the mean of 3 batches ± s.d.

Table S3. Composition in percentage by weight, of biloparticles used in the preformulation study.

Biloparticle system	Tristeari n	Bile acid	Miglyol 812	Poloxamer 188 (solution 2.5%w/w)
BilopS1	4.8	0.2	0	95
BilopS2	4.6	0.4	0	95
BilopS4	4.2	0.8	0	95
BilopS6	3.8	1.2	0	95

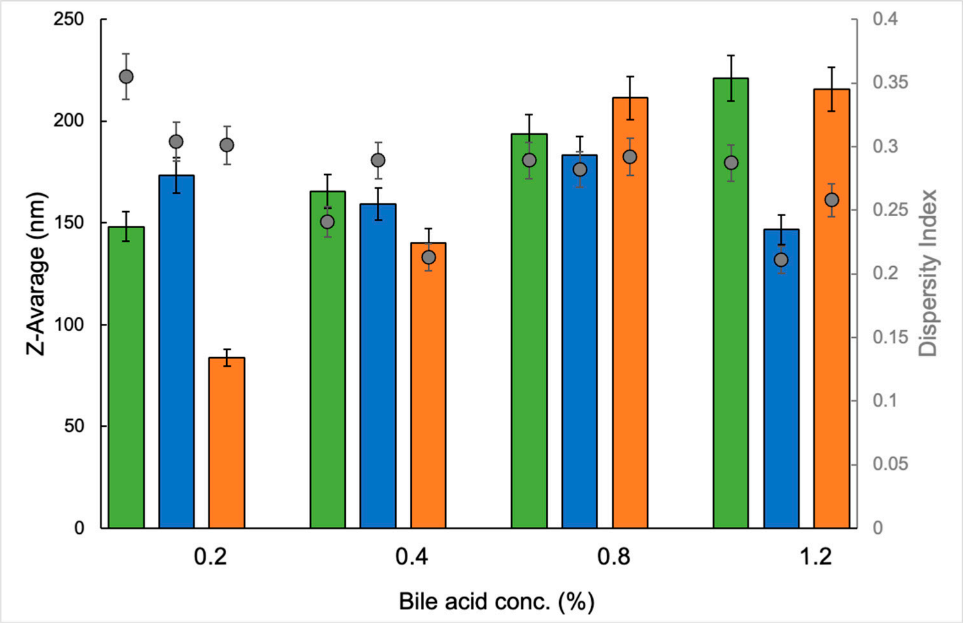

Figure S2. Mean diameter (Z-Average) (a) and Polydispersity (PdI) (b) after production of BilopS_C (green), BilopS_U (blue), BilopS_T (orange) with increasing concentration of bile acids as determined PCS. Each value is the average of 2 batches ± s.d.

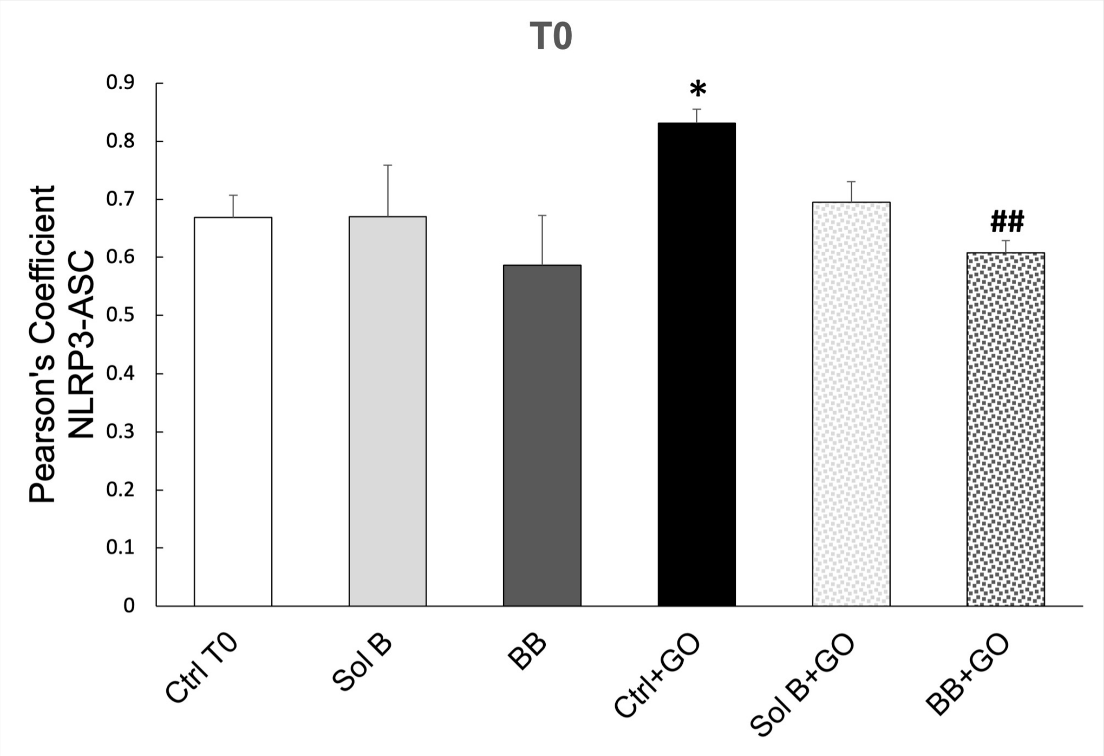

Figure S3. Colocalization expression levels of NLRP3 and ASC (Pearson's correlation coefficient) in CaCo2 cells treated with formulations containing budesonide and exposed to 0.5 U/ml of GO for 1 hour. The colocalization expression levels was quantified by using ImageJ software. Data are the results of the averages of at least three different experiments by 2-way ANOVA followed by Tukey's post-hoc comparison test. *p<0.05 ; ## p<0.01; (* respect Ctrl and # respect Ctrl+GO).
